# A Primer for Microbiome Time-Series Analysis

**DOI:** 10.3389/fgene.2020.00310

**Published:** 2020-04-21

**Authors:** Ashley R. Coenen, Sarah K. Hu, Elaine Luo, Daniel Muratore, Joshua S. Weitz

**Affiliations:** ^1^School of Physics, Georgia Institute of Technology, Atlanta, GA, United States; ^2^Woods Hole Oceanographic Institution, Marine Chemistry and Geochemistry, Woods Hole, MA, United States; ^3^Daniel K. Inouye Center for Microbial Oceanography: Research and Education, University of Hawaii, Honolulu, HI, United States; ^4^Interdisciplinary Graduate Program in Quantitative Biosciences, Georgia Institute of Technology, Atlanta, GA, United States; ^5^School of Biological Sciences, Georgia Institute of Technology, Atlanta, GA, United States

**Keywords:** microbial ecology, time-series analysis, marine microbiology, inference, clustering, periodicity, code:R, code:matlab

## Abstract

Time-series can provide critical insights into the structure and function of microbial communities. The analysis of temporal data warrants statistical considerations, distinct from comparative microbiome studies, to address ecological questions. This primer identifies unique challenges and approaches for analyzing microbiome time-series. In doing so, we focus on (1) identifying compositionally similar samples, (2) inferring putative interactions among populations, and (3) detecting periodic signals. We connect theory, code and data via a series of hands-on modules with a motivating biological question centered on marine microbial ecology. The topics of the modules include characterizing shifts in community structure and activity, identifying expression levels with a diel periodic signal, and identifying putative interactions within a complex community. Modules are presented as self-contained, open-access, interactive tutorials in R and Matlab. Throughout, we highlight statistical considerations for dealing with autocorrelated and compositional data, with an eye to improving the robustness of inferences from microbiome time-series. In doing so, we hope that this primer helps to broaden the use of time-series analytic methods within the microbial ecology research community.

## 1. Introduction

Microbiomes encompass biological complexity from molecules to genes, metabolisms, and community ecological interactions. Understanding this complexity can be difficult due to domain- or location- specific challenges in sampling and measurement. The application of sequencing technology has revolutionized almost all disciplines of microbial ecology, by allowing researchers the opportunity to access the diversity, functional capability, evolutionary history, and spatiotemporal dynamics of microbial communities rapidly and at a new level of detail (Huse et al., 2008; Caron, 2013). Increasingly it is now possible to sample at the time-scale at which those processes occur, resulting in the collection of microbiome time-series data. While such high-resolution sampling opens new avenues of inquiry, it also presents new challenges for analysis (McMurdie and Holmes, 2014; Weiss et al., 2016, 2017; Widder et al., 2016; Knight et al., 2018).

One of the first challenges in analyzing microbiome data is to categorize sequences in terms of taxa or even “species” (Konstantinidis et al., 2006; Caron and Hu, 2019). Many methods have been developed to perform this categorization (Blaxter et al., 2005; Konstantinidis and Tiedje, 2005; Huse et al., 2008; Mende et al., 2013; Sunagawa et al., 2013; Eren et al., 2014; Katsonis et al., 2014; Mahé et al., 2015; Varghese et al., 2015; Roux et al., 2016; Callahan et al., 2017; Luo et al., 2017). Particular choices used to define species-level units may alter downstream estimations of diversity and other parameters of interest (Youssef et al., 2009; Kim et al., 2011; Hu et al., 2015). Indeed, even the procedures for estimating common diversity parameters are impacted by the properties of read count data (Willis, 2019). However, some definition of taxa is often necessary for characterizing the composition of microbial communities. In this primer, we use the term *taxon* to denote approximately species-level designations, such as operational taxonomic unit (OTU) or amplicon sequence variant (ASV).

Once sequences have been categorized to approximate species-level groups, the interpretation of their read count abundances is accompanied by assumptions that violate many standard parametric statistical analyses. For example, zero reads from a sample mapping to a particular taxon is commonplace in microbiome sequence results, yet it typically remains unclear if a zero indicates evidence of absence (e.g., taxon not present in sample, incapable of transcribing a gene) or absence of evidence (e.g., below detection, inadequate sequencing depth) (Paulson et al., 2013; Weiss et al., 2017). In addition, sequence data is compositional, and therefore does not include information on absolute abundances (Gloor et al., 2017). As a result, compositional data has an intrinsic negative correlation structure, meaning that the increase in relative abundance of one community member necessarily decreases the relative abundances of all other members (Silverman et al., 2017).

**Figure 1:**
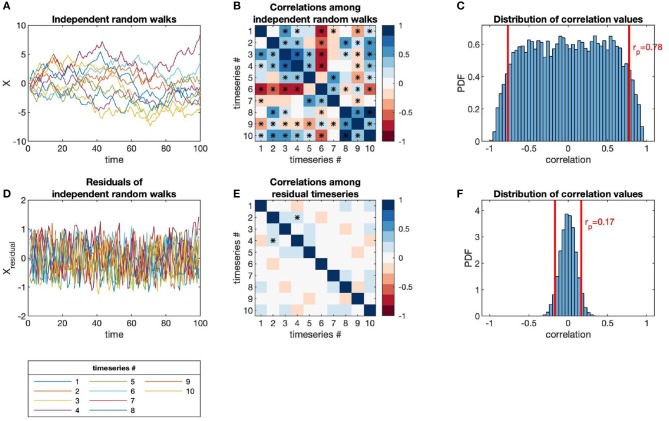
Independent random walks yield apparently significant correlations (when evaluated as independent pairs) despite no underlying interactions, in contrast to residuals (i.e., point-to-point differences). **(A)** Time-series of independent random walks, *x*_*i*_(*t*). **(B)** Correlation structure of independent random walks. **(C)** Distribution of correlation values for an ensemble of independent random walks, with *p*-value = 0.05 marked (red lines). **(D)** Time-series of the residuals of independent random walks, i.e., Δ*x*_*i*_(*t*) = *x*_*i*_(*t* + Δ*t*) − *x*_*i*_(*t*). **(E)** Correlation structure of residual time-series. **(F)** Distribution of correlation values for the same ensemble as **(C)** but taken between the residual time-series, with *p*-value = 0.05 marked (red lines).

The issues of categorization and sampling depth apply to all kinds of microbiome data sets. In particular, temporal autocorrelation presents an additional complexity to microbiome time-series, in that each observation is dependent on the observations previous to it in time. Autocorrelation also precludes the use of many standard statistical techniques, which assume that observations are independent. In Figure 1, we show how autocorrelation leads to high incidences of spurious correlations among independent time-series.

Complex microbiome data demand nuanced analysis. In this paper, we provide a condensed synthesis of principles to guide microbiome time-series analysis in practice. This synthesis builds upon and is complementary to prior efforts that established the importance of analyzing temporal variation for understanding microbial communities (e.g., Faust et al., 2015). Here, we introduce core statistical methods for microbiome time-series analysis as a starting point and suggest further reading on other possible methods. Our process is described in detail via several code tutorials at https://github.com/WeitzGroup/analyzing_microbiome_timeseries that include analytic tools and microbiome time-series data, and provide a software skeleton for the custom analysis of microbiome time-series data. These tutorials include the basics of discovering underlying structure in high-dimensional data via statistical ordination and divisive clustering, non-parametric periodic signal detection in temporal data, and model-based inference of interaction networks using microbiome time-series.

## 2. Methods

### 2.1. Overview of Tutorials

**Figure 2:**
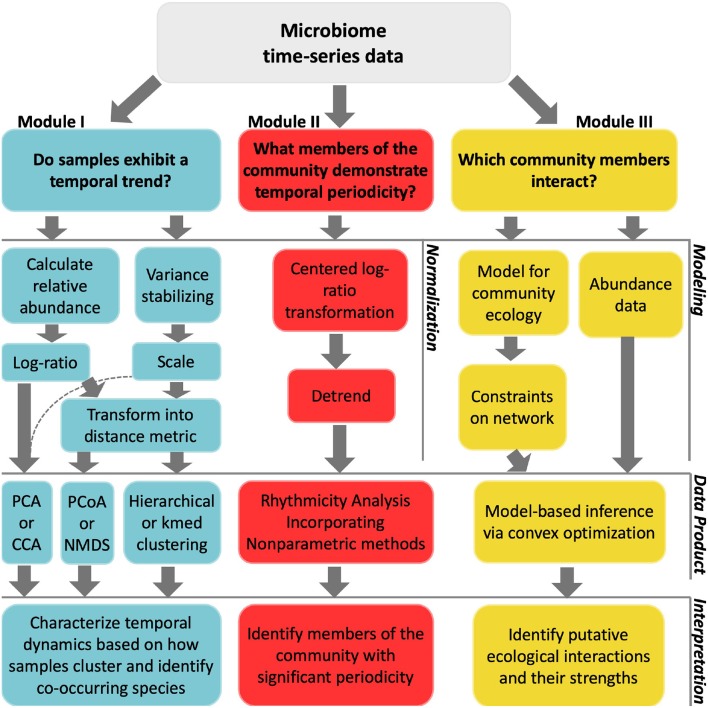
Workflow of techniques implemented in each module. The top layer considers questions of interest for a particular study. In the second layer, data normalizations are listed as implemented in module I and module II. For module III, we use synthetic data and instead list modeling inputs. The third layer shows the analytical techniques used in this primer, which we note is not exhaustive. These techniques provide some insight into the initial question asked, as described in the fourth layer.

We describe three distinct categories of time-series analyses: clustering, identifying periodicity, and inferring interactions. For each category, we demonstrate analyses that answer an ecologically motivated question (Figure 2). Each tutorial emphasizes normalization methods specifically developed for the analysis of compositional data. Each tutorial also addresses challenges related to multiple hypothesis testing, overdetermination, and measurement noise. Interactive, self-contained tutorials that execute the workflows described in the manuscript are available in R and Matlab https://github.com/WeitzGroup/analyzing_microbiome_timeseries.

### 2.2. Dataset Sources

For modules I and II, time-series data are derived from an 18S rRNA gene amplicon data set from Hu et al. (2018), in which samples were collected at 4 h intervals for a total of 19 time points (Lagrangian sampling approach). Input data are in the form of sequence count tables, where samples are represented as columns and each row is a taxonomic designation (OTU or transcript ID) with sequence counts or read coverage abundance per taxon (here we use “taxon” as shorthand). The code in each of these modules can be customized for use on other data, although for the purposes of analyzing any temporal-scale variability, samples must be taken at a frequency sufficiently shorter than the temporal scale of interest (e.g., daily temporal variability requires sub-daily sampling, seasonal temporal variability requires sub-seasonal sampling).

For module III, time-series data are simulated from a synthetic microbial community, for which the “true” network is known. The techniques in this module can be applied to time-series data as has been done in a handful of studies (Mounier et al., 2008; Stein et al., 2013; Fisher and Mehta, 2014; Marino et al., 2014; Dam et al., 2016; Jover et al., 2016; Ovaskainen et al., 2017; Xiao et al., 2017; Faust et al., 2018; Venturelli et al., 2018).

### 2.3. Normalization

#### 2.3.1. Log-Ratio Transformations

Microbiome data tend to have three properties: (1) they are sum-constrained (all reads sum to the sequencing depth), (2) they are non-negative, and (3) they are prone to heteroskedasticity (the variance of the data is not equal across its dynamic range). These attributes of microbiome data violate some underlying assumptions of traditional statistical techniques. Transforming microbiome data into log-ratios (Aitchison, 1983) can mitigate these problems by stabilizing variance and distributing values over all real numbers, as well as mitigating statistical bias related to sequencing protocols (McLaren et al., 2019).

The simplest log-ratio transformation requires selecting some particular focal variable/taxon in the composition, dividing all other variables in each sample by the abundance of the focal taxon, and taking the natural logarithm. Mathematically:

(1) $$\begin{array}{l} LR_{i}=ln\left(x_{i}\right)-ln\left(x_{focal}\right) \end{array}$$

This kind of log-ratio transformation eliminates negative constrained covariances, but all variables become relative to the abundance of an arbitrary focal taxon. Instead of selecting a focal taxon, the *Centered Log-Ratio Transformation* constructs ratios against the geometric average of community abundances (Egozcue et al., 2003).

(2) $$\begin{array}{l} CLR_{i}=ln\left(x_{i}\right)-\frac{1}{n}\overset{n}{\underset{k=1}{\sum }}ln\left(x_{k}\right) \end{array}$$

This transformation retains the same dimensionality as the original data, but is also still sum constrained:

(3) $$\begin{array}{l} \overset{n}{\underset{k=1}{\sum }}CLR_{k}=\overset{n}{\underset{k=1}{\sum }}\left(ln\left(x_{k}\right)-\frac{1}{n}\overset{n}{\underset{k=1}{\sum }}ln\left(x_{k}\right)\right) \end{array}$$

(4) $$\begin{array}{l} \overset{n}{\underset{k=1}{\sum }}CLR_{k}=\overset{n}{\underset{k=1}{\sum }}ln\left(x_{k}\right)-\frac{n}{n}\overset{n}{\underset{k=1}{\sum }}ln\left(x_{k}\right) \end{array}$$

(5) $$\begin{array}{l} =0 \end{array}$$

Log-based transformations require some caution when dealing with data sets with large numbers of zeros, namely because the logarithm of zero is undefined. To overcome this problem, implementations usually employ some pseudocount method, i.e., adding a small number to all observations to make the log of zero observations calculable. Adding a pseudocount disproportionately affects rare taxa, where the magnitudes of differences between samples may be similar to the magnitude of the added pseudocount and therefore obscured (Tsilimigras and Fodor, 2016).

#### 2.3.2. Z-Score Transformation

Another transformation that converts data from counts to a continuous real-valued number is the z-score transformation, achieved by applying this relationship:

(6) $$\begin{array}{l} z_{i}=\frac{x_{i}-\mu_{x}}{\sigma_{x}} \end{array}$$

where *x*_*i*_ is an observation, μ_*x*_ is the mean of population *x*, and σ_*x*_ is the standard deviation of *x*. Often, μ_*x*_ and σ_*x*_ are estimated by the sample mean and standard deviation. The z-score is how far, in terms of number of standard deviations, a given observation is from the sample mean Cheadle et al. (2003). Of note, this transformation places variables of different magnitudes on a scale with the same range.

#### 2.3.3. Variance Stabilizing Transformation

Log-ratio-based transformations in microbiome applications broadly serve the purpose of making the data more compatible with statistical methods that assume continuous/real-valued data and errors with equal variances. Such transformations are necessary because of the heteroscedasticity of sequence count data. A different approach to circumvent heteroscedastic data is to directly estimate a function which describes how the variance in the data increases as a function of the mean. Alternatively, it is possible to use a variance-stabilizing transformation, e.g., as implemented by the DESeq2 software package (Love et al., 2014). While the variance-stabilizing transformation is similar to a log transformation in the case of large counts, it is better suited to deal with zeros and does not rely on a pseudocount.

#### 2.3.4. Distance Metric

Multivariate microbiome data is not necessarily easy to summarize or visualize in two or three dimensions. Therefore, to summarize and explore data, we want to recapitulate the high-dimensional properties of the data in fewer dimensions. Such low-dimensional representations are distance-based. A distance matrix is obtained by applying a distance metric to all pairwise combinations of observations. For example, given data matrix *X*, the Euclidean distance between observations *X*_*i*_ and *X*_*j*_ is:

(7) $$\begin{array}{l} d{\left(X\right)}_{ij}=\sqrt{{\left(x_{i}-x_{j}\right)}^{2}} \end{array}$$

Different metrics measure distance using different attributes of the data [for comprehensive reviews of ecological distance metrics we recommend (Kuczynski et al., 2010; Buttigieg and Ramette, 2014)]. For example, only presence/absence of different community members is used to calculate Jaccard distance (Jaccard, 1912) and unweighted Unifrac (Lozupone and Knight, 2005), which also takes into account phylogenetic relationships between taxa. These metrics can be calculated on count data without transformation, and capture changes in the presence of rare taxa. On the other hand, Euclidean distance emphasizes changes in relative composition. Weighted Unifrac distance incorporates phylogenetic information as well as changes in relative abundances. Euclidean distance performed on log-ratio transformed data is analogous to Aitchinson's distance (Aitchison et al., 2000), which is recommended for the analysis of the difference of compositions.

In addition to distance metrics, sample-to-sample difference can also be compared by dissimilarities, such as the Bray-Curtis dissimilarity, which is defined between sample *i* and sample *j* as:

(8) $$\begin{array}{l} BC_{ij}=1-\frac{2\sum_{k=1}^{n}\min\left(s_{i,k},s_{j,k}\right)}{\sum_{k=1}^{n}s_{i,k}+\sum_{k=1}^{n}s_{j,k}} \end{array}$$

where *n* is the total number of unique taxon observed between both samples, and *s*_*i, k*_ is the abundance of taxon *k* in sample *i*. Bray-Curtis is widely used in ecological studies to measure differences in community composition (Bray and Curtis, 1957). A dissimilarity score of 0 means the two samples had identical communities, and a dissimilarity score of 1 means the two samples had no taxa in common. However, Bray-Curtis dissimilarity does not obey the triangle inequality (Gower and Legendre, 1986), which means that multivariate methods that assume distance matrices as input (e.g., NMDS) may yield uninterpretable results. For example, two samples that each have a Bray-Curtis dissimilarity of 0.05 from a third sample may have a Bray-Curtis dissimilarity of 1 from each other.

### 2.4. Ordination

#### 2.4.1. Covariance-Based Ordination

Statistical ordination can be used to explore multivariate microbiome data. An ordination is a transformation that presents data in a new coordinate system, e.g., making high-dimensional data visualizable in two or three dimensions. Principal Components Analysis (PCA) is a method which selects this coordinate system via the eigen decomposition of the sample covariance matrix, i.e., which is equivalent to solving the factorization problem:

(9) $$\begin{array}{l} Q_{m\times m}=U_{m\times m}D_{m\times m}U_{m\times m}^{T}. \end{array}$$

Here, *Q* is the sample by sample covariance matrix, *D* is a diagonal matrix containing the eigenvalues of *Q*, and *U* is a matrix of the eigenvectors associated with those eigenvalues. For PCA, the eigenvectors (or principal axes) are interpreted as new, uncorrelated variables, which are an orthogonal linear combination of the original *m* variables (Hotelling, 1933). Each of the eigenvalues corresponds to one of the eigenvectors and refers to its magnitude, which is proportional to the amount of variance in the data explained by that eigenvector. To plot a PCA, we select a subset of eigenvectors with the largest associated eigenvalues, apply the linear combination of variables contained in those eigenvectors to each observation, and then plot the observations with the resulting coordinates. Importantly, basic PCA relies on a least-squares approach for solving a linear model with the observed variables, which poorly models heteroscedastic non-negative data, such as taxon sequence counts. Non-linear PCA (Kramer, 1991) is one extension of PCA that can discover more sophisticated correlation structure between observed variables.

Principal Coordinates Analysis (PCoA), based on PCA, is another technique that allows for more flexibility in ordination modeling (Buttigieg and Ramette, 2014; Gloor et al., 2017). PCoA, on the other hand, uses the same procedure as PCA, except on a sample by sample distance matrix is decomposed instead of the sample covariance matrix (Borcard and Legendre, 2002), using the statistical properties of the distances instead of the original observed data. The choice of distance metric allows for the implementation of PCoA on either transformed (in which distance, such as euclidean may be suitable) or raw count (in which distance, such as Jaccard or unweighted Unifrac may be suitable) microbiome data. For both PCA and PCoA, scaling the data, for example with a z-score transformation, is recommended so that no one variable disproportionately influences the ordination (Holmes and Huber, 2019).

#### 2.4.2. Non-metric Multidimensional Scaling

Non-metric Multidimensional Scaling (NMDS) is an alternative ordination method which forces data to be projected into a pre-specified number of dimensions (Kruskal, 1964). NMDS projects high-dimensional data into a lower-dimensional space such that all pairwise distances between points are preserved. To implement NMDS, we solve the optimization problem:

(10) $$\begin{array}{l} \hat{X^{\prime }}=\text{arg min}||d\left(X\right)-d\left(X^{\prime }\right){||}_{2} \end{array}$$

where *X* is the original data matrix and *X*′ is the data in the lower-dimensional space. Here *d* is a distance metric (see Distance section). Because the sum of pairwise distances is the quantity being minimized by NMDS, this method is strongly affected by outliers, so data should be examined for outliers prior to NMDS ordination. Additionally, unlike PCA and PCoA, where the new sample coordinates are directly related to the measured variables, NMDS coordinates have no meaning outside of their pairwise distances. Another important difference between NMDS and PCA is that the NMDS is enforced to fit the ordination to a fixed number of dimensions, which means the projection is not guaranteed to be a good fit. *Stress* (Kruskal, 1964) is the quantification of how well the NMDS projection recapitulates the distance structure of the original data:

(11) $$\begin{array}{l} Stress=\sqrt{\frac{\sum {\left(d\left(X\right)-d\left(X^{\prime }\right)\right)}^{2}}{\sum d{\left(X\right)}^{2}}} \end{array}$$

The closer the stress is to 0, the better the NMDS performed.

#### 2.4.3. Clustering

Clustering defines relationships between individual data points, identifying a collection of points that are more similar to each other than members of other groups. Many clustering algorithms have been developed for the analysis of time series data (comprehensively reviewed in Liao, 2005). These algorithms include hierarchical methods, such as agglomerative clustering and k-medoids (McMurdie and Holmes, 2014; Gülagiz and Sahin, 2017), topological methods, such as self-organizing maps (Kohonen, 1990; Kavanaugh et al., 2014),and density-based methods, such as the DBSCAN algorithm (Khan et al., 2014). As a working example, we implement two types of hierarchical distance-based clustering algorithms, the partitioning about medoids (PAM or k-medoid) algorithm (Kaufman and Rousseeuw, 2009), and hierarchical agglomerative clustering (Murtagh, 1985). A hierarchical clustering method is one which works by partitioning the data into groups with increasingly similar features. The number of groups to divide the taxa into is determined prior to calculation, which begs the question: how many groups? This question can be quantitatively assessed using several indices. A clustering algorithm can be implemented using a range of possible numbers of clusters, and then comparison of these indices will indicate which number has a high degree of fit without over-fitting. These indices can also be used to help choose between clustering algorithms.

One such index is sum of squared differences, which is related to the total amount of uniformity in all clusters, defined as LaTeX error this align should read:

(12) $$\begin{array}{l} SSE=\overset{n_{\text{clusters}}}{\underset{k=0}{\sum }}\overset{n_{\text{members}}}{\underset{i=0}{\sum }}{\left(\overset{\text{Cluster member}}{\overset{︷}{x_{i,k}}}-\overset{\text{Cluster center}}{\overset{︷}{c_{k}}}\right)}^{2} \end{array}$$

A common heuristic to identifying an optimal number of clusters is to plot SSE vs. *k* and look for where the curve “elbows,” or where the decrease slows down (Liu et al., 2010; Gülagiz and Sahin, 2017) (see clustering tutorial).

Another way to evaluate the efficacy of clustering is via the Calinski-Harabasz index (Calinski and Harabasz, 1974), which is the ratio of the between-cluster squared distances to the within-cluster squared differences (Liu et al., 2010):

(13) $$\begin{array}{l} CH=\frac{\frac{B\left(x\right)}{k-1}}{\frac{W\left(x\right)}{n-k}} \end{array}$$

where *B*(*x*) is the between cluster sum of square differences, *W*(*x*) is the within cluster sum of square differences, *n* is the number of taxa, and *k* is the number of clusters. This index accounts for the number of clusters the data are partitioned into as well as the overall variation in the data as a whole. A large value of *CH* indicates that the between-cluster differences are much higher than the average differences between the dynamics of any pair of taxa in the data, so a maximum value of *CH* indicates maximum clustering coherence.

The “Silhouette width” is another index which allows for fine-scale examination of the coherence of individual taxon to their cluster. Silhouette width is therefore helpful for identifying outliers in clusters (Liu et al., 2010). The silhouette width for any given clustering of data is calculated for each taxon by taking the ratio of the difference between that taxon's furthest in-cluster neighbor and nearest out-of-cluster neighbor to the maximum of the two, such that

(14) $$\begin{array}{l} SW_{i}=\frac{\overset{\text{sum square diff out of cluster}}{\overset{︷}{min\left(d\left(x_{i},x_{j\notin C}\right)\right)}}-\overset{\text{sum square diff in cluster}}{\overset{︷}{max\left(d\left(x_{i},x_{j\in C}\right)\right)}}}{max\left(min\left(d\left(x_{i},x_{j\notin C}\right)\right),max\left(d\left(x_{i},x_{j\in C}\right)\right)\right)} \end{array}$$

where *C* is all taxa in the cluster, and *d* is the sum square difference operator. The widths can range from −1 to 1. Silhouette widths above 0 indicate taxa which are closer to any of their in-cluster neighbors than any out-of-cluster taxa, so having as many taxa with silhouette widths above 0 as possible is desirable. Any taxon with particularly low silhouette widths compared to the rest of their in-cluster neighbors should be investigated as potential outliers.

### 2.5. Periodicity Analysis

Periodicity analysis reveals whether or not a signal exhibits a cyclical periodic change in abundance. Approaches to identifying periodic signals include parametric methods and non-parametric methods. The multi-taper method is an example of a parametric method, which uses autoregression to find periodic signals in low signal-to-noise data (Mann and Lees, 1996) (for a software implementation in R https://cran.r-project.org/web/packages/ssa/index.html). Other examples of parametric methods include harmonic regression (Yang and Su, 2010; Ottesen et al., 2014), methods based on frequency spectral decomposition (Yang et al., 2011), and a widely used (Aylward et al., 2017; Hughes et al., 2017; Wilson et al., 2017; Hu et al., 2018) non-parametric method, “Rhythmicity Analysis Incorporating Non-parametric methods” (RAIN) (Thaben and Westermark, 2014).

The RAIN method identifies significant periodic signals given a pre-specified period and sampling frequency. RAIN then conducts a series of Mann-Whitney *U* tests [rank-based difference of means (Mann and Whitney, 1947)] between time-points in the time-series over the course of one period. For example, one such series of tests might answer the question: are samples at hours 0, 24, 48 higher in rank than the samples at hours 4, 28 (Hotelling, 1933). Then, the sequence of ranks is examined to determine if there is a consistent rise and fall about a peak time. For this procedure to work, RAIN relies on the assumption that time-series are stationary, or have the same mean across all sampled periods. One way to normalize microbiome time-series to better fit this assumption is detrending, or regression normalization, which removes longer-term temporal effects, such as seasonality. A first approximation of non-stationary linear processes can be made by taking the linear regression of all time-points with time as the independent variable, then subtracting this regression from the time-series. This operation stabilizes the data to have a similar mean across all local windows.

In order to assess periodicity for an entire microbial community, we may conduct many hypothesis tests. The more tests that are performed at once, the higher the probability of finding a low *p*-value due to chance alone (Streiner, 2015). Some form of multiple testing correction is therefore encouraged. False Discovery Rate (FDR) based methods are recommended for high-throughput biological data over more stringent Familywise Error Rate corrections (Noble, 2009; Glickman et al., 2014). The method employed here is the Benjamini-Hochberg step-up procedure (Benjamini and Yekutieli, 2001) (for graphical demonstration see the “periodicity” tutorial in the associated software package). *P*-values are ranked from smallest to largest, and all null hypotheses are sequentially rejected until test *k* where:

(15) $$\begin{array}{l} p_{k}\geq \frac{k}{m}\alpha \end{array}$$

where *m* is the total number of tests conducted, and α is the desired false discovery rate amongst rejected null hypotheses. Alternative *p*-value adjustment methods designed for sequencing data have been proposed (Conneely and Boehnke, 2007) which take into account correlation between tests, although simulations (Stevens et al., 2017) demonstrate that for moderate effect sizes, methods, such as Benjamini-Hochberg generally control false discoveries as expected, if not slightly more conservatively.

### 2.6. Inferring Interactions

#### 2.6.1. Model Specification of Ecological Dynamics

Inferring interactions using a model-based approach requires the specification of ecological (or eco-evolutionary) dynamics. Model specification requires extensive knowledge of the system of interest. Furthermore, models can be specified at diffierent levels of abstraction regarding taxonomic resolution (e.g. Storch and Šizling, 2008) and biological mechanisms (e.g. Vincenzi et al., 2016), leading to challenges in interpretability (Cao et al., 2017). Alternatively, data-driven identification of dynamical systems is an active area of research (e.g. Brunton et al., 2016; Mangan et al., 2016, 2017), providinga possible way forward when an appropriate model is not known *a priori*.

Currently, widely used models include some variation of Lotka-Volterra dynamics where each taxon is represented as a population whose abundances vary in time given density-dependent feedback with other populations (Mounier et al., 2008; Stein et al., 2013; Fisher and Mehta, 2014; Marino et al., 2014; Dam et al., 2016; Jover et al., 2016; Ovaskainen et al., 2017; Xiao et al., 2017; Faust et al., 2018; Venturelli et al., 2018). Here, we focus on a variant of this class of problem, i.e., virus-microbe dynamics.

The microbe-virus ecological dynamics are modeled via a system of differential equations

(16) $$\begin{array}{l} \dot{H_{i}}=r_{i}H_{i}\left(1-\frac{1}{K}\overset{N_{H}}{\underset{i^{\prime }}{\sum }}H_{i^{\prime }}\right)-H_{i}\overset{N_{V}}{\underset{j}{\sum }}M_{ij}\phi_{ij}V_{j} \end{array}$$

(17) $$\begin{array}{l} \dot{V_{j}}=V_{j}\overset{N_{H}}{\underset{i}{\sum }}M_{ij}\phi_{ij}\beta_{ij}H_{i}-m_{j}V_{j} \end{array}$$

where *H*_*i*_ and *V*_*j*_ denote the densities of host (i.e., microbe) type *i* and virus type *j* as they change over time. There are *N*_*H*_ host types and *N*_*V*_ virus types, each defined by their life history traits: growth rate *r*_*i*_ for host type *i*, decay rate *m*_*j*_ for virus type *j*, and a community-wide host carrying capacity *K*. The interactions between hosts and viruses are modeled as antagonistic infections culminating in the lysis (i.e., death) of the host cell and release of new viruses. For each pair host type *i* and virus type *j*, the infection is quantified by the interaction coefficient *M*_*ij*_, adsorption rate ϕ_*ij*_ and burst size β_*ij*_. The interaction coefficient is either 1 (the virus infects the host) or 0 (the virus does not infect the host) (Jover et al., 2013; Korytowski and Smith, 2017).

We randomly sample the life history traits and interaction parameters such that they are biologically plausible and guarantee local coexistence of all host and virus types (as described in Jover et al., 2016). We simulate the time-series of the resulting dynamical system using ODE45 in Matlab.

#### 2.6.2. Objective Function for Model-Based Inference

We seek the interaction network that minimizes the difference between observed dynamics in densities and those predicted by the dynamical model. We use the virus equations (Equation 17) to derive the objective function

(18) $$\text{min }{\left\|W-({\tilde{M}}^{T}-\vec{m})\left(\begin{array}{l} H\\ \vec{1} \end{array}\right)\right\|}_{2}\;+\lambda ||∼M{||}_{1}$$

(19) $$\begin{array}{l} \text{subject to }{\tilde{M}}_{ij}>0 \end{array}$$

(20) $$\begin{array}{l} m_{j}>0 \end{array}$$

where *W*_*jk*_ is the per-capita derivative estimate of virus type *j* at sampled time *t*_*k*_, *H*_*ik*_ is the density of host type *i* at sampled time *t*_*k*_, ${\tilde{M}}_{ij}^{T}=M_{ij}\phi_{ij}\beta_{ij}$ is the weighted infection coefficient between virus type *j* and host type *i* and *m*_*j*_ is the decay rate of virus type *j* (as described in Jover et al., 2016). We seek to estimate the unknown weighted infection network $\tilde{M}$, using sampled densities of hosts *H* and viruses *W* over time.

To prevent over-fitting, we introduce a hyper-parameter λ, which can be tuned to control the sparsity of the inferred network *M*. Other approaches can also be used to identifya balance between goodness of fit and model complexity, such as *k*-crossfold validation or information criterion (e.g. AIC). For an exampleof using *k*-crossfold validation, see Stein et al. (2013).

#### 2.6.3. Interaction Inference via Convex Optimization

In practice, we can solve the minimization problem (Equation 20) and infer the interaction network $\tilde{M}$ using convex optimization. Convex optimization is a well-developed technology for efficiently and accurately solving minimization problems of a particular form which are guaranteed to have a global minimum. Here, we use a freely available third-party software package for Matlab available for download at http://cvxr.com/cvx/ (Grant and Boyd, 2008, 2014) (also available for implementation in Python at https://www.cvxpy.org Diamond and Boyd, 2016; Agrawal et al., 2018). The details of implementation are described in Jover et al. (2016) and in the accompanying code tutorial.

In addition to convex optimization, there are many methods for inferring the interaction network, and dynamical systems parameters in general, from time-series. Two recent examples include MCMC fitting (Thamatrakoln et al., 2019; Zobitz et al., 2011) and Tikhonov regularization Stein et al. (2013).

## 3. Results and Discussion

### 3.1. Exploring Shifts in Daily Protistan Community Activity

The North Pacific Subtropical Gyre (NPSG) is widely studied as a model ocean ecosystem. Near the surface, the NPSG undergoes strong daily changes in light input. Abundant microorganisms in the NPSG surface community, such as the cyanobacteria *Prochlorococcus* and *Crocosphaera*, adapt metabolic activities, such as cell growth and division to particular times of day (Aylward et al., 2015; Ribalet et al., 2015; Wilson et al., 2017). However, the extent to which these daily cycles and the timings of particular metabolic activities extend to protistan members of the NPSG surface ecosystem remains less characterized. To this end, we examined an 18S rRNA gene diel dataset from a summer 2015 cruise sampled every 4 h for 3 days on a Lagrangian track near Station ALOHA (Hu et al., 2018). In this expedition, both rRNA and rDNA were sampled to explore differences in metabolic activity for particular community members at different times of day (Hu et al., 2016). Previous work (Hu et al., 2018) found shifts in the metabolically active protistan community, including phototrophic chlorophytes and haptophytes as well as parasitic Syndiniales.

**Figure 3:**
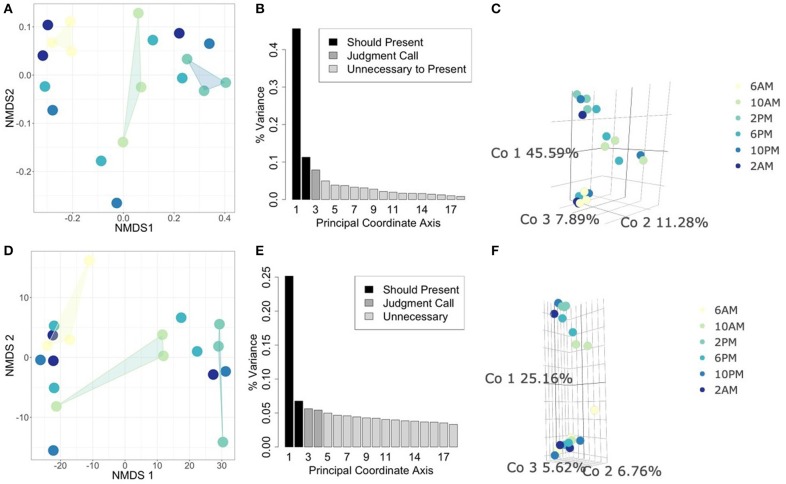
Comparing statistical ordination techniques for 18S community compositions across samples. (Top row) Ordinations using Jaccard distance for comparison of presence/absence of community members between samples. (Bottom row) Ordinations using Euclidean distance on isometric log-ratio transformed data. **(A,D)** Non-metric Multidimensional Scaling (NMDS) projection in two dimensions, arbitrary units. Convex hulls have been drawn to emphasize ordinal separation of 6 AM (yellow), 10 AM (light green), and 2 PM (teal) samples. **(B,E)** Scree plots for PCoA ordinations. Each bar corresponds to one axis of the PCoA, the height is proportional to the amount of variance explained by that axis. We decided the first 3 axes were necessary to summarize the data in these cases [explaining a total of **(B)** 64.76% and **(E)** 37.54% of the variance]. Shading of bars indicate our interpretations of which axes are important to show (black), which are unimportant (light gray), and which are intermediate cases (medium gray). **(C,F)** PCoA ordinations using the selected axes after scree plot examination. Each point is one sample, the color of the point indicates the time of day at which the sample was taken (colors correspond to NMDS projections).

In this analysis, we asked whether or not the metabolically active component of the microbial community is unique to different times of day. Therefore, we focused specifically on the 18S rRNA gene data as a proxy for overall functional activity of protistan taxa (Charvet et al., 2014; Hu et al., 2016; Xu et al., 2017). We used statistical ordination to explore underlying sample covariance. Samples that appear near each other in a statistical ordination have similar multivariate structure. In the clustering tutorial we present several methods for performing ordination, e.g., NMDS and PCoA (see Methods: Ordination). In Figures 3B,C, we construct a PCoA using Jaccard distance to emphasize changes in presence/absence of rRNA signatures, and find that the first 3 Principal Coordinates explain 64.76% of the variation amongst all samples. Samples from 2 PM and 6 AM strongly differentiate along the first coordinate axis, while samples at 10 AM settle between them. The ordination suggests that the taxa which are transcribing the 18S rRNA gene at 2 PM are fairly distinct from those transcribing at 6 AM, while 10 AM is intermediate between the two. We also constructed a corresponding NMDS ordination using the same distance matrix that we constrained to two dimensions. The pattern of separation between 2 and 6 PM is maintained in this projection, reinforcing its importance as an underlying structural feature of these data. Next, we constructed an additional PCoA ordination on the Euclidean distance matrix of isometric log-ratio transformed 18S rRNA counts (see clustering tutorial for implementation). We select the isometric log-ratio transformation to alleviate the constraint of compositionality and to scale the data to a similar range of magnitudes, making Euclidean distance a suitable choice of distance metric. As seen in the scree plot in Figure 3E, while the first Principal Coordinate explained about 25% of the variation between samples, the following four Principal Coordinates each explained around 5% of the variation. Despite the low proportion of total variance explained, strong separation emerges between 2 PM and 6 AM samples along the largest coordinate axis. This ordination qualitatively agrees with a corresponding NMDS ordination (Figure 3D) forced into two dimensions.

**Figure 4:**
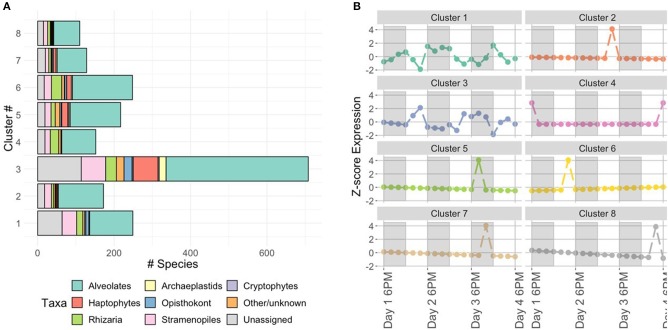
Characterization of protist clusters. **(A)** Cluster membership based on the phylum or class level protistan taxonomy. The “Other/unknown” category includes sequences with non-specific identity, such as “uncultured eukaryote” and “Unassigned” denotes sequences with no taxonomic hit (< 90% similar to reference database). **(B)** Representative taxon time-series for each cluster. Y-axis is z-score (see Methods: Normalizations), so a value of 0 corresponds to mean expression level. White and shaded regions represent samples taken during the light (white) dark cycle (shaded).

Noting the differences in active community members between 2 PM and 6 AM, we identified co-occurring taxa by clustering their temporal dynamics after variance-stabilization and scaling normalizations (see clustering tutorial for discussion). Based on comparisons of sum squared errors and the CH index introduced in Methods, we opted to divide the OTUs into eight clusters (Figure 4 for composition and representative temporal signature, tutorial for details on cluster selection). After comparing cluster evaluation metrics for hierarchical agglomerative clustering and a k-medoids algorithm, we conducted this clustering with k-medoids (see clustering tutorial for implementation). This method allows us to identify the time-series of the median taxon for each cluster as a representative shape for the cluster's temporal dynamics. We observe 2 PM peaks associated with clusters 2, 3, 6, and 8 and increased nighttime expression levels in cluster 1. These temporal patterns coincide with those surmised during our exploratory ordination of the community sampled at each time point (where 2 PM and 6 AM samples formed distinct clusters, Figure 3). Upon closer inspection of cluster membership (bar plots in Figure 4A), we find cluster 3 contains 65/105 (62%) of haptophyte OTUs and 18/33 (55%) of archaeplastids, including members of chlorophyta.

These results suggest temporal niche partitioning within the complex protistan community, consistent with the findings of Hu et al. (2018). By clustering results with respect to temporal patterns, we were able to parse the complex community to reveal the identities of key taxonomic groups driving the observed temporal patterns. The taxonomic composition of cluster 3 was made up of haptophytes and chlorophytes. Photosynthetic chlorophytes have previously been found to be correlated with the light cycle (Poretsky et al., 2009; Aylward et al., 2015) and the temporal pattern found in Hu et al. (2018) was similar to the standardized expression level (Figure 4B), as was the inferred relative metabolic activity of haptophytes.

### 3.2. Identifying Protists With Diel Periodicity in 18S Expression Levels

The metabolic activity of microbes is a critical aspect of the basis of marine food webs (Karl, 2002). In the euphotic zone, microbial populations are inherently linked to the light cycle as the energy source for metabolism. Identifying diel patterns in protists is particularly interesting due to widespread mixotrophy, where a mixotroph may ingest prey during periods of limiting inorganic nutrients or light (Nygaard and Tobiesen, 1993; Finkel et al., 2009; McKie-Krisberg et al., 2015). Additionally, protistan species encompass a wide range of cell sizes, thus the synchronization of light among photoautotrophs may reflect species-specific differences in nutrient uptake strategies (Hein et al., 1995; Gerea et al., 2019). Based on the observation of sample differentiation between the middle of the day (2 PM) and dawn (6 AM) from exploratory ordination and clustering analyses described in 4.1, we further investigated the hypothesis that some protists may exhibit a 24-h periodicity in their 18S rRNA gene expression levels.

**Figure 5:**
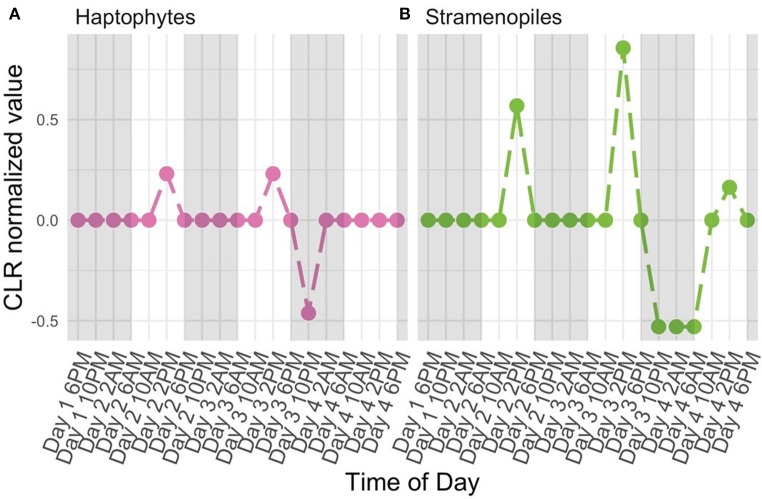
Centered Log Ratio (CLR)-transformed, detrended 18S rRNA gene levels (y-axes) over time (x-axes) for a subset of OTUs found to have significant diel periodicity (RAIN analysis). A value of 0 denotes the mean expression level for a given OTU. Included OTUs belong to the **(A)** Haptophyte and **(B)** Stramenopile groups. White and shaded regions represent samples taken during the light (white) dark cycle (shaded).

The high-resolution nature of the sequencing effort in this study enabled us to ask which members of the protistan community had 24-h periodic signals. Following normalization (CLR, Equation 2) and detrending to center mean expression levels across the entire time series (see Periodicity tutorial and Methods: Periodicity Analysis), we used RAIN to assess the periodic nature of each OTU over time. Results from RAIN analysis reported *p*-values for each OTU at the specified period as well as estimates of peak phase and shape. The null hypothesis tested by RAIN is that the observations do not consistently increase, then decrease (or vice-versa) once over the course of a period. Rejecting the null hypothesis, then, asserts a time-series has one peak during the specified period. To determine which OTUs were found to have significant periodicity we rejected the null hypothesis at 5% FDR level (Equation 13). Figure 5 illustrates examples of two protistan OTUs with significant diel periodicity, a haptophyte and stramenopile. Trends in CLR normalized values for each OTU indicated that there was a repeated and temporally coordinated relative increased in the metabolic activity of both taxa at 2 PM (Figure 5). Both groups have previously been found to respond to day-night environmental cues, which is also supported by Hu et al. (2018).

Identities of OTUs found to have significant diel periodicity included taxa with known phototrophic and/or heterotrophic feeding strategies. This suggests that taxa with diel changes in metabolic activity may be responding to light or availability of prey. More specifically, several known phototrophs or mixotrophs, including dinoflagellates, haptophytes, and stramenopiles were found to have significant diel periodicity. Interestingly, there were a number of OTUs identified as belonging to the Syndiniales group (Alveolates) which are obligate parasites. Diel rhythmicity among these parasites suggests that they may be temporally coordinated to hosts that also have a periodic signal, which includes dinoflagellates.

### 3.3. Inferring Interactions in a Synthetic Microbial Community

The goal of an inference method is to quantify ecological interactions between pairs of populations or taxonomic designation of interest. The result of such analysis is an interaction network for the community of interest. In the context of microbial communities, “interaction” can be broadly defined and include, for example, direct competition for a nutrient, toxin-mediated attacks, or cooperation via exchange of secondary metabolites. Besides pairwise interactions between microbes, other interactions may be of interest, such as higher-order interactions [e.g., three-way microbial exchanges (Fisher and Mehta, 2014; Bairey et al., 2016; Grilli et al., 2017)], pressures from other trophic levels (e.g., grazers, viruses), or driving via environmental variables (e.g., antibiotics, nutrient flux). Inferring interaction networks is a challenging task, in part due to autocorrelation inherent in time-series data. Time-series which are highly autocorrelated appear correlated with one another, even when there is no underlying causal relationship (see Figure 1). This leads to high false-positive rates of inferred interactions, particularly for correlation-based inference methods (Kurtz et al., 2015; Weiss et al., 2016; Coenen and Weitz, 2018; Carr et al., 2019; Hirano and Takemoto, 2019; Mainali et al., 2019; Thurman et al., 2019).

Model-based inference methods are built from dynamical models of microbial community ecology. As such, temporal variation and structure is incorporated into any model-based inference framework, accounting for potentially difficult statistical properties, such as autocorrelation. Model-based inference has been shown to perform favorably in *in silico* studies (Mounier et al., 2008; Stein et al., 2013; Fisher and Mehta, 2014; Marino et al., 2014; Dam et al., 2016; Jover et al., 2016; Ovaskainen et al., 2017; Xiao et al., 2017; Faust et al., 2018; Venturelli et al., 2018). Major challenges remain for implementing model-based inference in practice, including requirements of high time-resolution data and a detailed understanding of the biological and ecological mechanisms at play in order to correctly specify the underlying model. Futhermore, evaluating accuracy of inferred networks remains dificult, in part because difierent networks can produce similar patterns of ecological dynamics (Cao et al., 2017). Despite challenges, model-based inference has shown potential to accurately infer interaction networks in a computationally efficient and scalable manner (see one such application in Stein et al., 2013).

**Figure 6:**
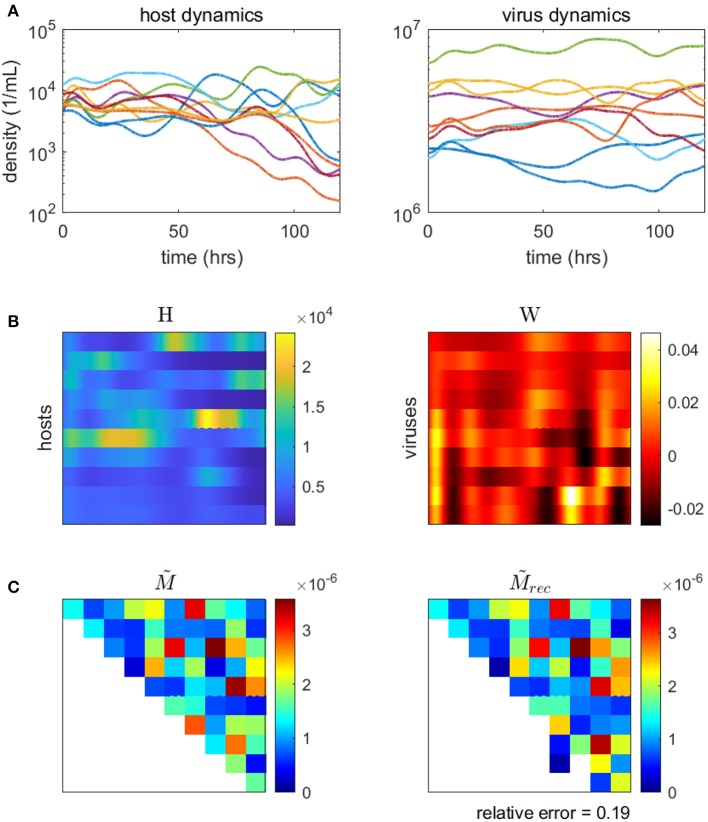
Inferring the microbe-virus infection network from time-series data for a 10 by 10 synthetic microbe-virus community. **(A)** Simulated host (left) and virus (right) densities over time. **(B)** Host densities (left, H) and transformed virus differences (right, W), for input into the objective function (Equation 20). **(C)** The original “ground-truth” interaction network (left) and the reconstructed network (right). In the interaction matrix, the rows denote hosts, the columns represent viruses, and the colors denote the scaled intensity of interactions (where white denotes no interaction).

Here, we demonstrate the use of a model-based inference method on a synthetic microbial community with viruses (methods and code adapted from Jover et al., 2016). We use a synthetic community so that the inferred network can be compared to the original, “ground-truth” network. Using our model for microbe-virus ecological dynamics (Equation 17), we simulate population time-series of the community over the course of several days. We sample the simulated time-series to use as data inputs into the minimization problem (Equation 20), from which we estimate the weighted microbe-virus infection network $\tilde{M}$. Simulated time-series, data inputs, original and reconstructed networks are shown in Figure 6). As shown, the reconstructed network closely resembles the original, with only minor quantitative differences (i.e., in the strengths of the interactions). We caution that the choice (and parameterization) of ecological dynamics is critical to developing a model-based approach, for alternative examples see Mounier et al. (2008), Stein et al. (2013), Fisher and Mehta (2014), Marino et al. (2014), Dam et al. (2016), Jover et al. (2016), Ovaskainen et al. (2017), Xiao et al. (2017), Faust et al. (2018), and Venturelli et al. (2018).

## 4. Conclusion

The aim of this primer was to integrate analytic advances together to serve practical aims, so that they can be transferred for analysis of other high resolution temporal data sets. Conducting high-resolution temporal analyses to understand microbial community dynamics has become more feasible in recent years with continued advances in sequence technology. Accordingly, specific statistical considerations should be taken into account as a precursor for microbiome analysis. In this primer, we summarized challenges in analyzing time-series data and present examples which synthesize practical steps to manage these challenges. For further reading on the topics addressed here, we recommend: normalizations and log-ratios (Egozcue et al., 2003; Silverman et al., 2017), distance calculations (Willis and Martin, 2018), clustering (Kurtz et al., 2015; Martin-Platero et al., 2018), statistical ordination (Morton et al., 2017; Ren et al., 2017), regression (Martin et al., 2019), vector autoregression (Opgen-Rhein and Strimmer, 2007), periodicity detection (Ernst and Bar-Joseph, 2006), general best practices (Holmes and Huber, 2019), and an in-depth review of multivariate data analysis (Buttigieg and Ramette, 2014). For inferring interactions from time-series, model-based inference approaches have significant potential (Mounier et al., 2008; Stein et al., 2013; Fisher and Mehta, 2014; Marino et al., 2014; Dam et al., 2016; Jover et al., 2016; Ovaskainen et al., 2017; Xiao et al., 2017; Faust et al., 2018; Venturelli et al., 2018). Although correlation-based methods have been widely used for inferring interactions, recent literature suggests that correlation-based methods are poor indicators of interaction (Weiss et al., 2016; Coenen and Weitz, 2018; Carr et al., 2019; Hirano and Takemoto, 2019; Mainali et al., 2019; Thurman et al., 2019). Other model-free methods, such as Granger causality (Mainali et al., 2019) and cross-convergent mapping (Sugihara et al., 2012), may be useful alternatives for inference although care should be taken that data do not violate the methods' assumptions (McCracken and Weigel, 2014; Baskerville and Cobey, 2017). In closing, we hope that the consolidated methods and workflows in both R and Matlab help researchers from multiple disciplines advance the quantitative *in situ* study of microbial communities.

## Data Availability Statement

For the 18S rRNA gene-based survey, data originated from Hu et al. (2018). The raw sequence data can also be found under SRA BioProject PRJNA393172. Code to process this 18S rRNA tag-sequencing data can be found at https://github.com/shu251/18Sdiversity_diel and quality checked reads and final OTU table used for downstream data analysis is available (10.5281/zenodo.1243295), as well as in the GitHub https://github.com/WeitzGroup/analyzing_ microbiome_timeseries.

## Author Contributions

AC, SH, EL, DM, and JW conceptualized the work. SH provided the data for analysis. AC, DM, and JW designed the methods and analyses. SH and DM wrote the code for the clustering and periodicity tutorials. AC wrote the code for the inference tutorial. AC, SH, EL, DM, and JW co-wrote the manuscript. All authors approved the manuscript.

## Conflict of Interest

The authors declare that the research was conducted in the absence of any commercial or financial relationships that could be construed as a potential conflict of interest.
